# Self-Controlled Cleaving Method for Silicon DRIE Process Cross-Section Characterization

**DOI:** 10.3390/mi12050534

**Published:** 2021-05-08

**Authors:** Dmitry A. Baklykov, Mihail Andronic, Olga S. Sorokina, Sergey S. Avdeev, Kirill A. Buzaverov, Ilya A. Ryzhikov, Ilya A. Rodionov

**Affiliations:** 1 FMN Laboratory, Bauman Moscow State Technical University, 105005 Moscow, Russia; m_andronic@bmstu.ru (M.A.); soros@bmstu.ru (O.S.S.); avdeevss@student.bmstu.ru (S.S.A.); kirillbuz@bmstu.ru (K.A.B.); nanocom@yandex.ru (I.A.R.); irodionov@bmstu.ru (I.A.R.); 2 Dukhov Automatics Research Institute, (VNIIA), 127055 Moscow, Russia; 3 Institute for Theoretical and Applied Electromagnetics RAS, 125412 Moscow, Russia

**Keywords:** Bosch process, DRIE of Silicon, MEMS, cross-section cleaving

## Abstract

Advanced microsystems widely used in integrated optoelectronic devices, energy harvesting components, and microfluidic lab-on-chips require high-aspect silicon microstructures with a precisely controlled profile. Such microstructures can be fabricated using the Bosch process, which is a key process for the mass production of micro-electro-mechanical systems (MEMS) devices. One can measure the etching profile at a cross-section to characterize the Bosch process quality by cleaving the substrate into two pieces. However, the cleaving process of several neighboring deeply etched microstructures is a very challenging and uncontrollable task. The cleaving method affects both the cleaving efficiency and the metrology quality of the resulting etched microstructures. The standard cleaving technique using a diamond scriber does not solve this issue. Herein, we suggest a highly controllable cross-section cleaving method, which minimizes the effect on the resulting deep etching profile. We experimentally compare two cleaving methods based on various auxiliary microstructures: (1) etched transverse auxiliary lines of various widths (from 5 to 100 μm) and positions; and (2) etched dashed auxiliary lines. The interplay between the auxiliary lines and the etching process is analyzed for dense periodic and isolated trenches sized from 2 to 50 μm with an aspect ratio of more than 10. We experimentally showed that an incorrect choice of auxiliary line parameters leads to silicon “build-up” defects at target microstructures intersections, which significantly affects the cross-section profile metrology. Finally, we suggest a highly controllable defect-free cross-section cleaving method utilizing dashed auxiliary lines with the stress concentrators.

## 1. Introduction

**Figure 1:**
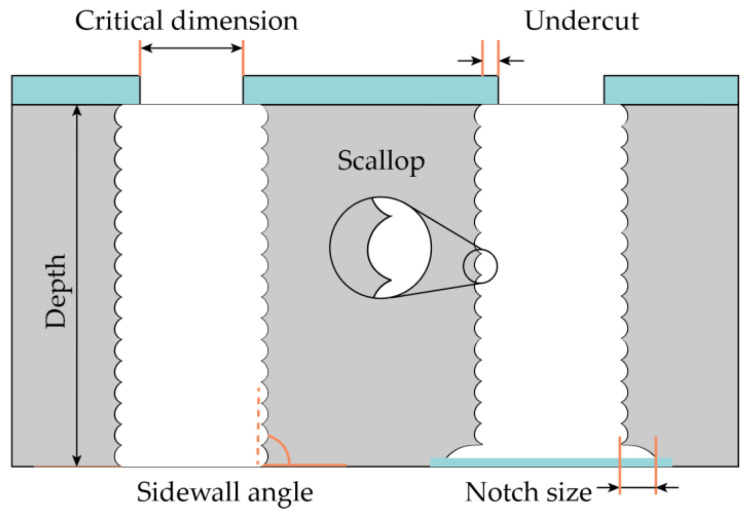
Schematic view of cross-section profile after the Bosch process silicon etching.

Micro-electro-mechanical systems (MEMS) are versatile microdevices that are widely used in many areas of human life from scientific research to industry [1,2,3]. Silicon microtechnology based on microelectronic techniques [4] is the key mass production fabrication method for creating microsystems. Compared to planar integrated circuits, MEMS 3D devices are always characterized by unique design (structures size and shape), individual materials stack, and device-friendly fabrication technology. Usually, highly sensitive state-of-the-art MEMS devices require forming a high aspect ratio (more than 20) microstructures with precisely controlled profiles [5,6]. The Bosch process is one of the most popular processes for deep anisotropic reactive ion etching of silicon, which has many modifications and allows one to make high aspect ratio microstructures [7,8,9,10]. The main Bosch process optimization parameters are the elimination of etching defects (“micrograss”, wall erosion, and wall roughness), etching rate nonuniformity, profile angle control over the wafer, etc. In addition, one should pay attention to ARDE (Aspect Ratio Dependent Etching) and microloading effects i.e., the influence of microstructure topology (different widths and shape) on the etching process. A process-dependent feature of the Bosch process is rippled sidewalls of the structures (Figure 1), which are unacceptable for some devices, for example, when creating through-silicon vias (TSV), and these require additional processing [11,12,13,14].

**Figure 2:**
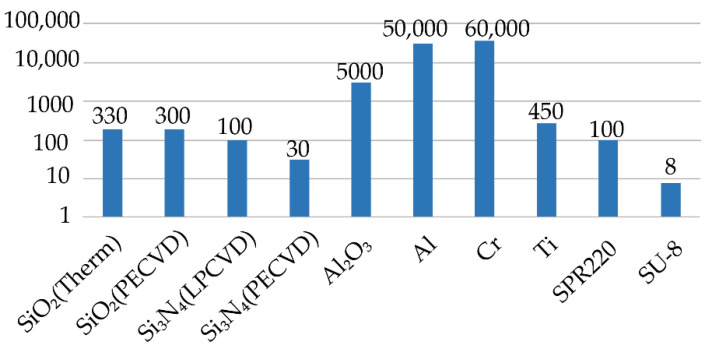
Basic protective masks for the Bosch process and their selectivity.

The Bosch process deep silicon etching (hundreds of microns deep) with a high aspect ratio (>20) requires an appropriate choice of protective mask. There are several materials compatible with dry etching processes and suitable for this purpose (Figure 2): silicon dioxide and silicon nitride, metal masks, and photoresists [15,16,17,18,19,20,21,22,23,24]. Particular mask choice can depend on not only selectivity but relies on available equipment and materials in the laboratory. From this point of view, thermal silicon dioxide is one of the most commonly used materials for MEMS devices. On the one hand, it provides high selectivity, which makes it possible to fabricate complex designs and get microscale resolution. On the other hand, silicon dioxide depositions and processing methods are well studied and do not require large resources for optimization [25,26,27]. Moreover, silicon dioxide is not susceptible to overheating during etching compared to photoresist masks and does not have a micromasking effect opposite to using metal hard masks.

**Figure 3:**
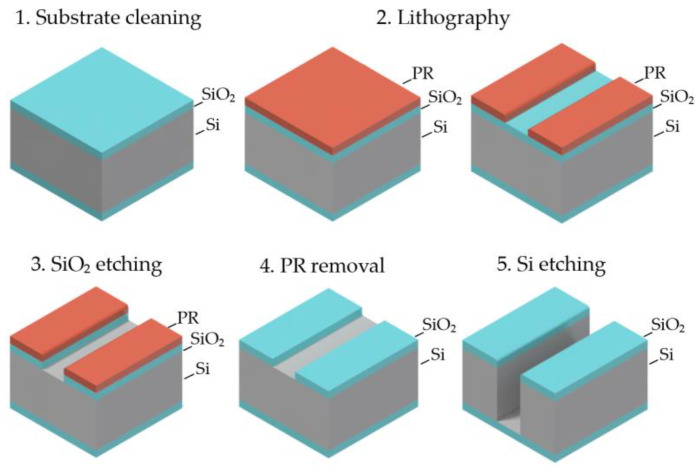
The sequence of operations for creating trenches in silicon.

There are is a standard sequence of basic operations to fabricate the required device topology on a silicon substrate, which are surface preparation, photoresist spin coating, photolithography, photoresist development, SiO_2_ etching, photoresist removal, and silicon etching by the Bosch process (Figure 3). Optimizing process windows for each technology step in this route allows selecting the device-dependent fabrication process and fixing design rules for a specific MEMS device type.

One of the challenging issues of the Bosch process optimization is the complex interplay between device design (topology dimensions and shape) and etch parameters. Experimental studies show that the etching rate primarily depends on the trench width; nevertheless, trench length and shape also affect the etching mechanism, but to a smaller degree [28,29]. Furthermore, for higher aspect ratios due to changes in etching mixture supply and removal conditions, the etching rate decreases with increasing depth (ARDE effect) [30]. To etch microstructures with different dimensions and shape, one has to design an etching process with overetch (for through etching of silicon), which needs a lager etching process window and higher selectivity. The overetching step in the process can lead to a notching effect, which takes place when the reactive mixture interacts with the bottom dielectric surface (SiO_2_) of the substrate [31]. There are several ways to eliminate this defect [32,33,34], but it requires additional process optimization and several additional steps.

Another worth mentioning aspect of the Bosch process is the design pattern density impact on etching itself. According to the paper [35], local pattern density (microloading effect) affects the etching rate within the radius of 4.5 mm decreasing it by 1.5% when the density increases by 10%. There is research [36] showing that with an increase of etchable area (fill factor) from 1% to 95%, the etching rate can change by a factor of 5. Moreover, profile parameters as well as etching rate distribution over the sample (in the center and at the edge) are also changed. On the other hand, there are papers [37,38] demonstrating a small impact of microloading effect compared to the influence of trench width and aspect ratio, which primarily determine the etching process results.

One can notice contradicting results and conclusions when analyzing etching process optimization studies. The reasons for these mismatches lie in an enormous amount of etching process parameters and definition of metrology methods for assessing the etching output characteristics: selectivity, uniformity, etching rate, profile angle, and set of test topologies. First, a set of test structures geometries has to be determined, which is necessary and sufficient to characterize the Bosch etching process. It should contain a set of single lines [34,39], dense lines, and more complex topological structures with different etching depths and pattern densities [40,41,42,43,44,45]. However, despite the wide scope of research forming the standard set of geometries for the Bosch test topology, there is no general approach to cleaving methods for these microstructures in order to get a controllable high-quality sample cross-section, which is critical for optimizing the Bosch deep-etching process. 

**Figure 4:**
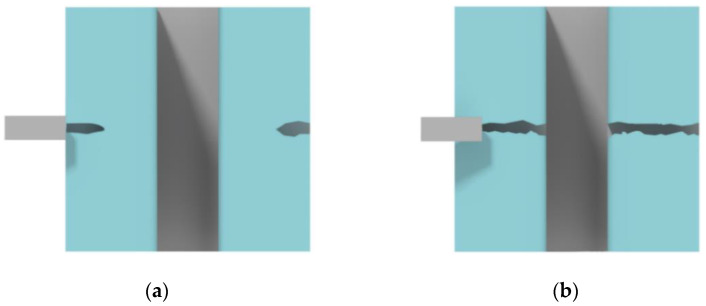
The standard approach for sample cleaving with high trenches in silicon: (**a**) Small scratch near the trench; (**b**) Long scratch crossing the trench.

There are several methods for the controlled cleaving of silicon substrates, for example, preliminary micro-groove formation with subsequent thermal stress cleaving using lasers [46,47,48]. On the other hand, cleaving can be performed by etching v-groove with the sequential use of a diamond scriber [49]. Both approaches involve wet etching at the first stage, which greatly complicates the overall cleaving technique. In addition, such methods can result in destroying microstructures of small sizes. Adding transverse auxiliary lines across all the microstructures is one of the possible solutions to get controlled cleavage [40], but it is unclear if these auxiliary lines affect the dimensions and etching process at intersections. One of the most commonly used methods for cleaving silicon substrates for inspection is diamond scribing across the crystallographic orientation of silicon. In this case, a stressed layer formed with the scriber (Figure 4a) ensures the cleaving direction. However, for deeply etched structures, short scratches at the edge of the substrate do not provide effective cleaving; since such structures tend to fracture, the cleaving direction tends to be uncontrolled. As the result, one needs to use long scratches through target microstructures for better control of the cleaving (Figure 4b), but microstructures metrology suffers.

**Figure 5:**
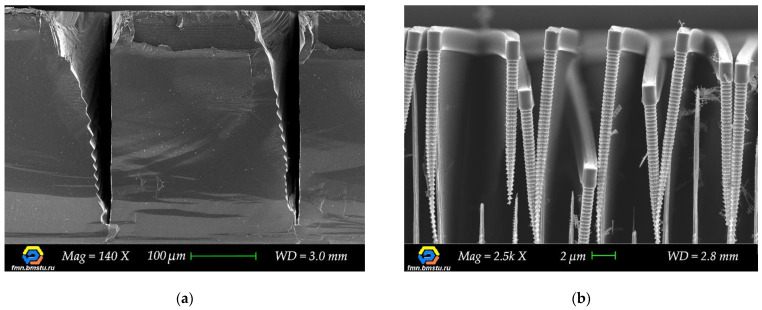
Scanning electron microscopy (SEM) images of cleaving defects: (**a**) Destruction of the upper part of the microstructure, which spreads downward due to the presence of a large number of stress concentrators; (**b**) Bending and destruction of 2-μm structures under their weight due to additional etching at the intersection with auxiliary lines.

Despite the relatively simple procedure of long scratch, the subsequent characterization of microstructures after the Bosch process becomes challenging. In most cases, particularly for micrometer-scale structures, their upper part is destroyed after diamond scribing with damage spreading downward due to a large number of stress concentrators (Figure 5a). That is why this cleaving method is not suitable for further cross-section analysis. Moreover, at higher local density (smaller pitch) of narrow microstructures, etched silicon structures destruction can be observed during splitting or etching of the sample (Figure 5b). Hence, the high-quality metrology method, which guarantees reproducible cleaving process and cross-section profile measurements for various types of microstructures, is required.

As mentioned above, adding transverse auxiliary lines that are etched simultaneously with test structures can be successfully used for the controlled cleaving technique [40]. However, there are a few unobvious points. It is worth noticing that cross-section measurements of target microstructures are performed directly at these intersections and can cause extremely big critical dimensions metrology errors. In this paper, we present our research of interplay between different auxiliary cleaving elements and target microstructures with dimensions from 2 to 50 μm. We experimentally compare two cleaving methods utilizing various auxiliary microstructures: (1) etched transverse auxiliary lines of various widths (from 5 to 100 μm) and positions crossing target microstructures; (2) etched dashed auxiliary lines. Based on this research, we propose a highly controllable defect-free cross-section cleaving method utilizing dashed auxiliary lines with the stress concentrators for reliable Bosch deep etching process optimization. 

## 2. Materials and Methods

In all the experiments, we used 25 × 25 mm^2^ substrates diced from 100 mm diameter p-type silicon wafers (10–20 Ω·cm) with the crystal orientation of <100>. Thermal oxide with a thickness of 4 μm was deposited to be used as a hard mask layer. To pattern the silicon dioxide layer, we used 4 μm thick Megaposit SPR220 photoresist. Pattern transfer processes were carried out using a Heidelberg Instruments (Heidelberg, Germany) μPG101 laser lithography system. Topology transferring to a protective mask (SiO_2_) through SPR220 photoresist was carried out using reactive ion etching in a CHF_3_/Ar gases using an Oxford PlasmaPro100 etcher. The inductively coupled plasma (ICP) etcher also implemented the Bosch process. Optical microscopy and field emission scanning electron microscopy were used to measure critical dimensions both from the top surface of the samples and cross-sections for the Bosch process quality control.

For deep anisotropic silicon etching, we used a three-stage Bosch process with steps of passivation, breakthrough, and etching. The breakthrough step was designed to remove the polymer predominantly from the bottom of structures characterized by a higher displacement to the substrate. C_4_F_8_ was chosen as the passivating gas, the main etching gas was SF_6_; for better removal of the polymer from the bottom of the trenches, O_2_ was added at the stage of breakthrough and etching. The operating temperature during the process was 5 °C. The key parameters of the deep reactive ion etching (DRIE) process are listed in Table 1.

**Figure 6:**
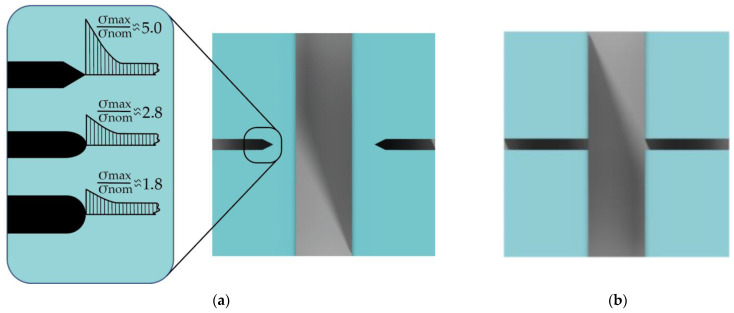
Two methods of forming auxiliary cleaving lines: (**a**) Etched dashed auxiliary line with the stress concentrators; (**b**) Transverse etched auxiliary crossing line.

Two main methods of creating auxiliary cleaving lines (Figure 6) for the Bosch process characterization were chosen to compare their influence on target microstructures with sizes from 2 to 50 μm. In the first and second cases, dashed and transverse auxiliary crossing lines were formed during the etch process, and then substrates were cleaved along these lines. The key difference between these cases is an absence of a direct intersection between auxiliary lines and target microstructures. We analyzed various designs of stress concentrators (Figure 6a) for dashed auxiliary cleaving lines. The degree of stress increase depends primarily on the type and shape of the auxiliary line. The greater the cross-sectional difference in the transition section and the sharper the transitions and undercuts, the higher the local maximum stress occurs [50]. We choose angular structure as the main shape because it provides up to 5 times higher maximum stress than the nominal one.

**Figure 7:**
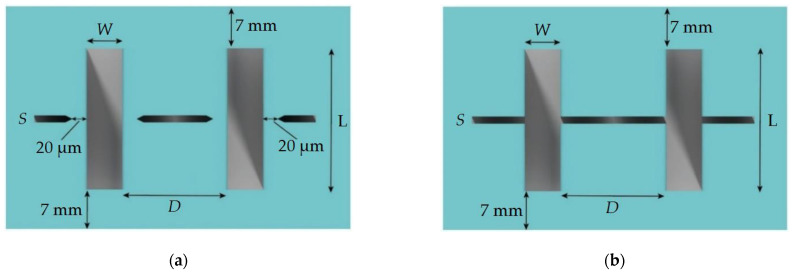
Typical dimensions of the test topology: (**a**) Dashed auxiliary lines; (**b**) Transverse crossing auxiliary lines.

In order to investigate the influence of the sample cleaving method, a test topology was developed, which include the set of lines (with widths (*W*) from 2 to 50 μm and lengths (L) of 1 mm) with ratio (width (*W*) to distance between lines (*D*)) of 1:1 and 1:10 for each standard size. For all the structures with dashed auxiliary lines, the line width and the distance from the line to the target microstructure was 20 μm (Figure 7a). For the structures with transverse crossing lines, the width of the auxiliary line (*S*) was chosen as 5, 50, and 100 μm (Figure 7b). In all cleaving methods, the distance from the edge of the substrate to the test lines was at least 7 mm. 

**Figure 8:**
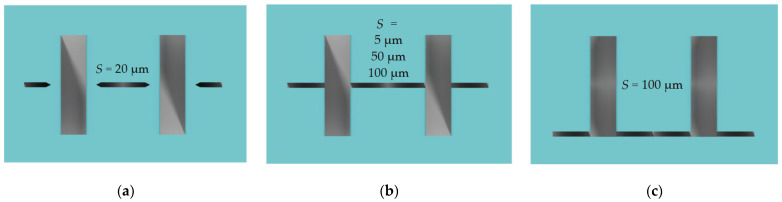
Test topology appearance: (**a**) Dashed auxiliary line (reference); (**b**) Transverse crossing auxiliary line in the center; (**c**) Transverse crossing auxiliary line on the edge.

In addition, we tried to vary the position of the auxiliary line relative to target microstructures (Figure 8c). A detailed list of the test structures is given in Table 2 and Figure 8.

We decided to set target microstructures with dashed auxiliary lines as a reference for comparison with other types of lines (Figure 8b,c) because they have no direct intersections with auxiliary lines (Figure 8a) and evidentially have no additional influence during the etching process. It should be noted that fixed values of the dashed auxiliary line width of 20 μm (*S*) and the distance to the microstructure were chosen. When selecting it, we were guided by the fact that auxiliary lines should be deep enough after etching and close enough to target microstructures to control the cleaving. On the other hand, it should be as narrow as possible to minimize its influence on the etching process of target microstructures.

We observed the destruction of 2 μm target lines with *W:D* = 1:1 ratio due to their long length (1000 μm), which is not always the case for real MEMS devices (Figure 5b). We found out that this destruction was caused by 2 μm structures weakness; they started bending under their weight and additional reaction with process gases mixture.

## 3. Results

In this study, we performed a series of Bosch deep etching experiments to compare the influence of cleaving methods on the quality and values of measured out process parameters. The etch rate (V) was calculated as the total etch depth per number of Bosch cycles. Selectivity was calculated as the ratio between silicon etching rates and silicon dioxide etching rates. The profile angle (A) demonstrates the structure profile, when A is less than 90, it tapers; when A is more than 90, it expands. 

Table 3 shows the data for target lines from 2 to 50 μm with *W:D* = 1:1 ratio, and Table 4—data for target lines with *W:D* = 1:10 ratio. The etching depth for 5 μm target lines was at least 250 μm, and for 50 μm target lines, it was at least 500 μm in all the experiments. That results in a maximum aspect ratio greater than 50 and a minimum aspect ratio of at least 10. 

Target microstructures with dashed auxiliary lines were chosen as a reference for comparison with other types of lines, as they have no direct intersections with auxiliary lines and have no additional treatment during etching.

**Figure 9:**
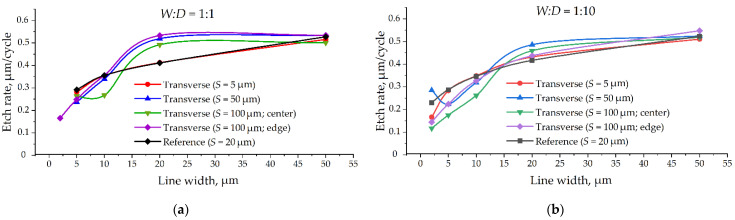
Etching rate depending on the target lines width: (**a**) For *W:D* = 1:1 aspect ratio; (**b**) For *W:D* = 1:10 aspect ratio.

A pretty general tendency of increasing the etching rate with increased target line width can be observed on the graphs (Figure 9) based on the tabular data (Table 3 and Table 4). One can easily notice a huge difference in data and curve character for the same target microstructures depends on the cleaving method.

**Figure 10:**
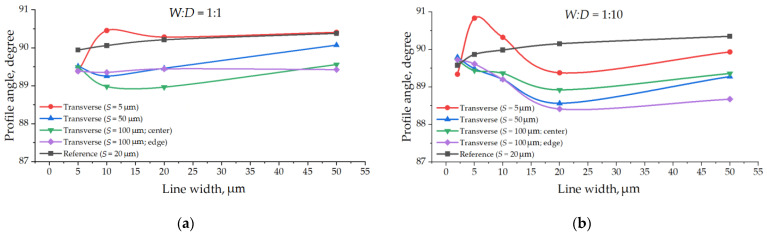
Profile angle depending on the target lines width: (**a**) For *W:D* = 1:1 aspect ratio; (**b**) For *W:D* = 1:10 aspect ratio.

In general, we can see no tendency for the target line’s width to influence its profile angle (Figure 10). However, one can observe a clear dependence of cleaving method on the profile angle, as the auxiliary transverse lines influence differently the target line profiles.

Figure 9 and Figure 10 demonstrate direct the evidence that the cleaving method can result in both quantitative and qualitative mistakes in Bosch deep etching process research. It is very important to estimate the influence of metrology techniques on Bosch deep etching process output parameters and carefully choose nondestructive auxiliary lines for efficient cleaving.

## 4. Discussion

### 4.1. Influence of the Auxiliary Cleaving Lines on the Etching Rate

**Figure 11:**
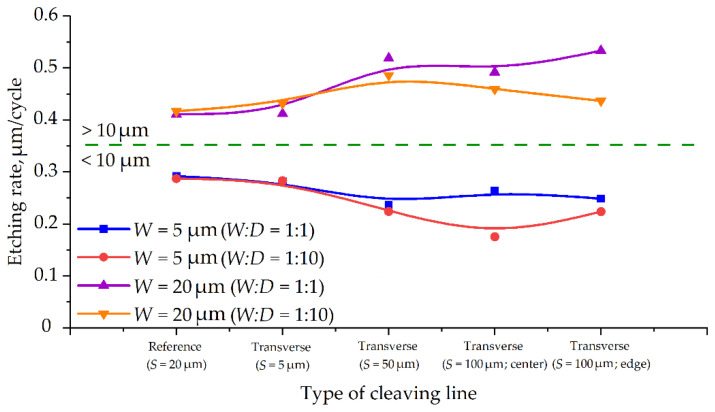
Etching rate change depending on cleaving auxiliary lines type and target lines width.

Based on the results obtained on etching rates for target microstructures with different types of auxiliary line intersection, we found out that for the sub-10 μm target lines etching rate becomes higher without intersection (dashed reference line, *S* = 20 μm). At the same time, for >10 μm target lines, the highest etching rate is observed when crossing with wide auxiliary lines (*S* = 50 μm and *S* = 100 μm) and the lowest, on the contrary, is observed for reference dashed auxiliary lines (*S* = 20 μm, Figure 11). 

In addition, for transverse crossing auxiliary lines, a steplike transition in etching rate is observed in the range from 10 to 20 μm (up to 85%), which indicates a qualitative change in the etching process. For target microstructures without intersection with auxiliary lines, the maximum etching rate change in the range from 10 to 20 μm is only about 15%.

### 4.2. Influence of the Auxiliary Cleaving Lines on Etching Behavior

**Figure 12:**
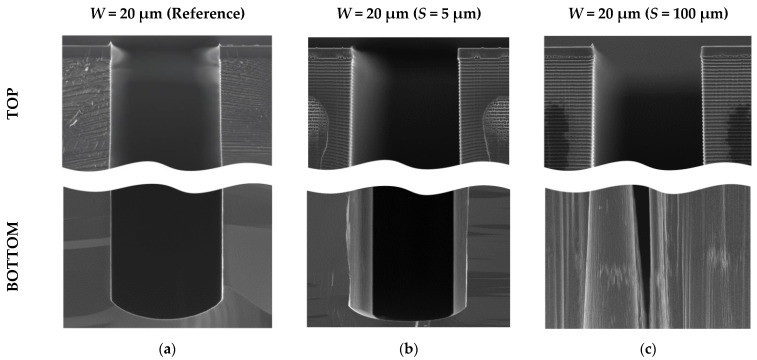
Twenty μm wide target lines profiles comparison for different cleaving lines: (**a**) Dashed auxiliary line (reference); (**b**) 5 μm wide transverse crossing auxiliary lines (*S* = 5 μm); (**c**) 100 μm wide transverse crossing auxiliary lines (*S* = 100 μm).

While analyzing a lot of target microstructures cross-sections with scanning electron microscopy (for different cleaving methods), we observed clear evidence and the source of change in etching behavior. We noticed that target lines with dashed auxiliary lines (reference, without intersection) have no profile narrowing during etching when the etching depth becomes bigger. Their shape was retained until the end of the etching. On the other hand, when switching to *S* = 5 µm transverse auxiliary lines, the “second” structure profile appeared (Figure 12b) at the intersection with the target structure, forming a narrower trench. For wider transverse auxiliary lines, for example, *S* = 100 μm, significant narrowing of the profile took place—up to complete stop of the etching process, which was observed for *W* < 20 μm in many cases (Figure 12c). 

**Figure 13:**
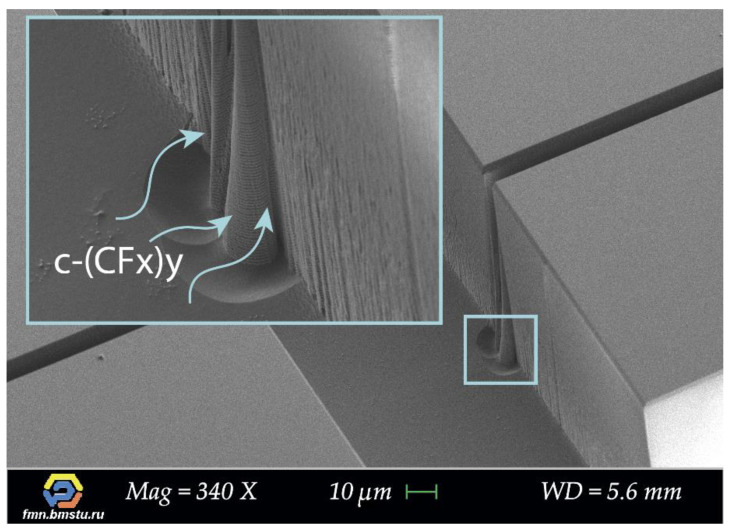
SEM images of “build-up” defects and etched groove formation in the area of intersection target microstructures and 20 μm wide transverse crossing auxiliary lines.

This change in etching behavior is associated with silicon “build-up” defects forming during the Bosch deep etching process. “Build-up” defects occur due to increased polymer formation at intersection areas, which prevents target microstructures etching and leads to the narrowing of trenches gradually. The reason is a local area of intersection, where additional geometry appears in the form of corners of target microstructures. Corners are subjected to additional action of ions on each side, which leads to more efficient polymer formation due to sticking coefficient increase [51,52,53]; moreover, the character of supply and removal of the gases mixture into the trench changes. Additionally, big grooves are formed at the bottom of the structure in the area of intersection, which may indicate local charge forming (on dielectric “build-up” defects) and etching particles deflection forming an additional etched area around (Figure 13).

**Figure 14:**
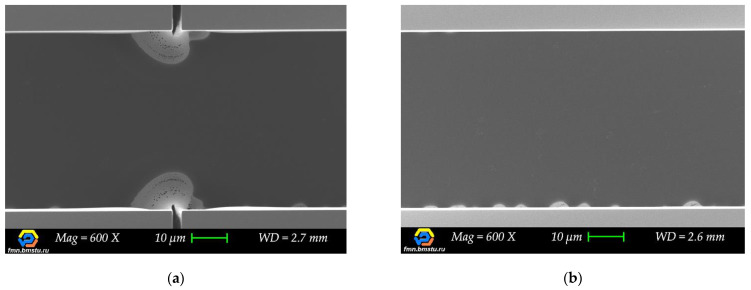
Top view SEM images of the intersection area: (**a**) Transverse crossing auxiliary lines; (**b**) Dashed auxiliary lines.

It is well known that narrowing defects of the target microstructures can be the result of non-optimal Bosch process parameters, but not the geometry features of auxiliary lines. However, in this case, a comparison of the lines with and without crossing auxiliary lines showed that in the intersection area without crossing, there are no “build-up” defects or it is much fewer (Figure 14).

**Figure 15:**
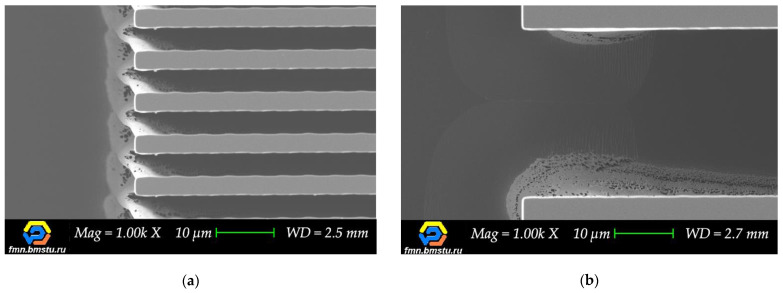
Polymerization at the intersections of transverse crossing auxiliary lines (S = 100 μm) with: (**a**) 5 μm wide target line; (**b**) 50 μm wide target line.

Thinking about industry applications, one can notice that there are no devices with straight lines only in topology. Therefore, when designing a test topology set or developing devices, it is important to precisely control intersection areas and any deviations of the shape from a straight line. Polymerization at the intersections during the Bosch deep etching process is the most critical for topologies with microscale critical dimensions (Figure 15).

### 4.3. Influence of the Position of the Auxiliary Cleaving Lines

We experimentally confirmed that the position of transverse crossing auxiliary lines (*S* = 100 μm) also affects the trench etching process. The etching rate becomes higher when target structures intersect transverse crossing auxiliary lines at the edge for both *W:D* = 1:1 and *W:D* = 1:10. At the areas where the array of target lines switches to single target lines, a significant narrowing of trenches is observed. This effect is the result of increased polymerization in the Bosch process due to a local decrease in structure density at the intersections with the constant supply of gas mixture at the same time. For topologies with a centered position of transverse crossing auxiliary lines (*S* = 100 μm), the profiles of target structures for *W:D* = 1:1 and *W:D* = 1:10 are almost the same.

### 4.4. Influence of the Auxiliary Cleaving Lines on the Profile Angle

**Figure 16:**
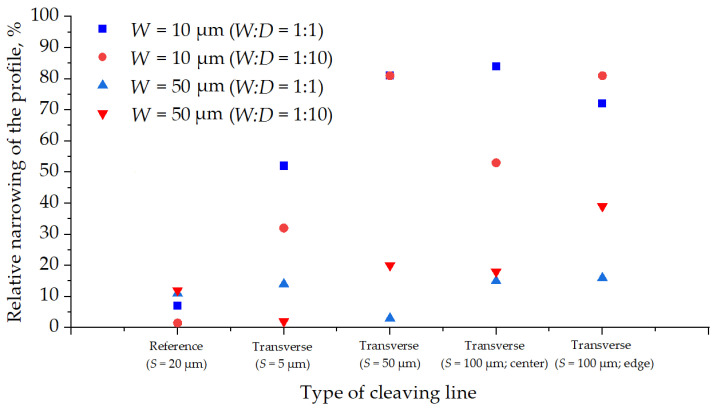
The relative narrowing of the profile per trench width depending on the type of cleaving line.

The influence of “build-up” defects on profile angle at the intersections of target structures with transverse crossing auxiliary lines is significant. Comparing the graphs (Figure 9 and Figure 10), one can see that on average, target structures with transverse crossing auxiliary lines are much narrower than structures without intersection. The graph (Figure 16) shows the relative narrowing of the structures profile per its width.

It should be noted that for sub-20μm trenches with transverse-crossing auxiliary lines, profile narrowing can reach up to 85% from the original trench width, and for structures wider than 20 μm, it can reach up to 40%. For lines without intersections, the maximum deviation does not reach more than 12% of the original trench width.

### 4.5. Influence of the Distance between Target Microstructures

For target microstructures without intersection (dashed auxiliary lines *S* = 20 μm), the maximum difference between the etching rates for *W:D* = 1:1 and *W:D* = 1:10 was 2.5% for *W* = 10 μm. The change in the profile angle for this type of target line was also minor. As the width of transverse crossing auxiliary lines increases (from *S* = 5 μm to *S* = 100 μm), the difference between single lines (*W:D* = 1:10) and an array of lines (*W:D* = 1:1) become bigger from 5% to 33%, which indicates a large contribution of line density to the etching pattern. It is also worth noting that for most types of intersecting structures (transverse crossing auxiliary lines), the etching rate is higher at *W:D* = 1:1. This phenomenon may be associated with a change in the distribution mechanism of the polymer. With an increase in the local density of trenches, the depletion of the active polymer mixture occurs, and consequently, the etching rate increases.

## 5. Conclusions

Various types of auxiliary lines for controlled cleaving of DRIE silicon substrate were analyzed and tested for target microstructures with a width from 2 to 50 μm and an aspect ratio of more than 10. The most common cleaving method utilizing crystallography orientated scratch features in silicon (using diamond scriber) is uncontrollable, not reproducible, and not accurate enough for many types of microstructures. Cleaving methods based on transverse auxiliary lines for splitting substrates with microstructures are much more reliable and accurate. In this article, we experimentally confirmed that the intersection areas of target microstructures with auxiliary lines lead to narrowing of the profile during the Bosch process due to a change in supply and removal flows of polymerizing gas mixture. The wider the auxiliary lines, the narrower the target structures at the intersections. According to our experiments, the best cleaving results for all types of target microstructures can be received with dashed auxiliary lines with sharp end-forming stress concentrators. This cleaving method makes it possible to control the characteristics of the sample without adding etching defects. The etching behavior of single target lines and dense target lines (arrays) changed in case of intersecting transverse crossing auxiliary lines but remained unchanged for the dashed auxiliary line.

## Figures and Tables

**Table 1:** Deep reactive ion etching (DRIE) parameters of the used recipe in this study.

Step	Inductively Coupled Plasma (ICP) (W)	Radiofrequency (RF) (W)	Pressure (mTorr)	Process Gases
Passivation	1200–1500	5	20	C_4_F_8_/SF_6_/O_2_ mixture
Breakthrough		50	30	
Etching		5	40	

**Table 2:** Description of the test structure dimensions.

Type of Cleaving Lines	Cleaving Lines Width (*S*), μm			Target Line Width (*W*), μm					Target Line Length (L), μm
Dashed auxiliary lines	20			2	5	10	20	50	1000
Transverse crossing auxiliary lines	5	50	100						

**Table 3:** Deep etching experimental results for target microstructures with aspect ratio *W:D* = 1:1.

Type of Cleaving Auxiliary Lines	Process Parameter	Target Lines Width				
		2 μm *	5 μm	10 μm	20 μm	50 μm
Dashed auxiliary lines (Reference) (*S* = 20 μm)	V, μm/cycle	-	0.292	0.356	0.411	0.527
	Selectivity	-	126	154	178	243
	A, degree	-	89.94	90.06	90.21	90.38
Transverse auxiliary lines (*S* = 5 μm)	V, μm/cycle	-	0.282	0.355	0.412	0.516
	Selectivity	-	122	153	178	223
	A, degree	-	89.39	90.45	90.28	90.41
Transverse auxiliary lines (*S* = 50 μm)	V, μm/cycle	-	0.237	0.340	0.519	0.533
	Selectivity	-	102	147	225	231
	A, degree	-	89.51	89.26	89.46	90.07
Transverse auxiliary lines (*S* = 100 μm, center)	V, μm/cycle	-	0.263	0.268	0.492	0.501
	Selectivity	-	114	116	213	217
	A, degree	-	89.49	88.98	88.96	89.56
Transverse auxiliary lines (*S* = 100 μm, edge)	V, μm/cycle	0.165	0.249	0.356	0.533	0.534
	Selectivity	72	108	154	231	231
	A, degree	-	89.38	89.35	89.44	89.43

* Target line with the width of 2 μm was broken.

**Table 4:** Deep etching experimental results for target microstructures with aspect ratio *W:D* = 1:10.

Type of Cleaving Line	Process Parameter	Target Lines Width				
		2 μm	5 μm	10 μm	20 μm	50 μm
Dashed auxiliary lines (Reference) (*S* = 20 μm)	V, μm/cycle	0.230	0.287	0.346	0.417	0.523
	Selectivity	93	116	140	168	211
	A, degree	89.58	89.87	89.98	90.15	90.35
Transverse auxiliary lines (*S* = 5 μm)	V, μm/cycle	0.167	0.283	0.349	0.432	0.510
	Selectivity	72	123	151	187	221
	A, degree	89.34	90.83	90.32	89.38	89.93
Transverse auxiliary lines (*S* = 50 μm)	V, μm/cycle	0.285	0.224	0.319	0.486	0.524
	Selectivity	123	97	138	210	227
	A, degree	89.80	89.49	89.20	88.57	89.28
Transverse auxiliary lines (*S* = 100 μm, center)	V, μm/cycle	0.117	0.175	0.262	0.459	0.519
	Selectivity	51	76	113	199	225
	A, degree	89.72	89.44	89.36	88.92	89.36
Transverse auxiliary lines (*S* = 100 μm, edge)	V, μm/cycle	0.145	0.224	0.327	0.437	0.548
	Selectivity	63	97	141	189	237
	A, degree	89.72	89.61	89.20	88.41	88.68

## References

[1] Castañer L. (2015). Understanding Mems: Principles and Applications.

[2] Zhou G., Lee C. (2017). Optical MEMS, Nanophotonics, and Their Applications.

[3] Choudhary V., Iniewski K.I. (2017). Mems: Fundamental Technology and Applications.

[4] Androniс M.M., Rodionov I.A., Tsvetkov Y.B. Digital design as a key approach to shortening MEMS development cycle. Proceedings of the ITM Web of Conferences.

[5] Bhardwaj J., Ashraf H., McQuarrie A. Dry silicon etching for MEMS. *Symp. Microstruct. Microfabr. Syst.*
**1997**, 1–13. http://luxor.quantumlabs.co/home/admin/QUANTUMFAB1/PUBLICATIONS/Macropore%20Formation/dry_si_etching.pdf.

[6] Rangelow I.W. (2003). Critical tasks in high aspect ratio silicon dry etching for microelectromechanical systems. J. Vac. Sci. Technol. A Vac. Surf. Films..

[7] Laermer F., Andrea U. (2019). MEMS at Bosch–Si plasma etch success story, history, applications, and products. Plasma Proc. Polym..

[8] Chang B., Jensen F., Hübner J., Jansen H. (2018). DREM2: A facile fabrication strategy for freestanding three dimensional silicon micro-and nanostructures by a modified Bosch etch process. J. Micromech Microeng..

[9] Westerman R., Martinez L., Pays-Volard D., Mackenzie K., Lazerand T. (2014). Deep silicon etching: Current capabilities and future directions. Micromach. Microfabr. Proc. Technol. XIX Int. Soc. Optics Photonics.

[10] Marty F., Rousseau L., Saadany B., Mercier B., Français O., Mita Y., Bourouina T. (2005). Advanced etching of silicon based on deep reactive ion etching for silicon high aspect ratio microstructures and three-dimensional micro-and nanostructures. Microelectron. J..

[11] Ranganathan N., Lee D.Y., Youhe L., Lo G.Q., Prasad K., Pey K.L. (2011). Influence of Bosch etch process on electrical isolation of TSV structures. IEEE Trans. Components Packaging Manuf. Technol..

[12] Gao F., Ylinen S., Kainlauri M., Kapulainen M. (2014). Smooth silicon sidewall etching for waveguide structures using a modified Bosch process. J. Micro/Nanolithogr. MEMS MOEMS.

[13] Fu J., Li J., Yu J., Liu R., Li J., Wang W., Chen D. (2018). Improving sidewall roughness by combined RIE-Bosch process. Mater. Sci. Semiconduct. Process..

[14] Mohammed Z.A.S., Olimpo M.A.S., Poenar D.P., Aditya S. (2017). Smoothening of scalloped DRIE trench walls. Mater. Sci. Semiconduct. Process..

[15] Etching B. A Deep Silicon RIE Primer Bosch Etching of Deep Structures in Silicon. *Power*
**2009**. https://www.nanofab.ualberta.ca/wp-content/uploads/2009/03/primer_deepsiliconrie.pdf.

[16] Shearn M., Sun X., Henry M.D., Yariv A., Scherer A., Grym J. (2010). Advanced plasma processing: Etching, deposition, and wafer bonding techniques for semiconductor applications. Semiconductor Technologies.

[17] Huber R., Conrad J., Schmitt L., Hecker K., Scheurer J., Weber M. (2003). Fabrication of multilevel silicon structures by anisotropic deep silicon etching. Microelectron. Eng..

[18] Bagolini A., Ronchin S., Bellutti P., Chistè M., Verotti M., Belfiore N.P. (2017). Fabrication of novel MEMS microgrippers by deep reactive ion etching with metal hard mask. J. Microelectromech. Syst..

[19] Rahiminejad S., Cegielski P., Abassi M., Enoksson P. (2016). A four level silicon microstructure fabrication by DRIE. J. Micromech. Microeng..

[20] Ganji B.A., Majlis B.Y. Deep trenches in silicon structure using DRIE method with aluminum as an etching mask. Proceedings of the 2006 IEEE International Conference on Semiconductor Electronics.

[21] Aydinoglu F., Saffih F., Dey R.K., Cui B. (2017). Chromium oxide as a hard mask material better than metallic chromium. J. Vac. Sci. Technol. B Nanotechnol. Microelectron. Mater. Process. Meas. Phenomena..

[22] Grigoras K., Sainiemi L., Tiilikainen J., Säynätjoki A., Airaksinen V., Franssila S. (2007). Application of ultra-thin aluminum oxide etch mask made by atomic layer deposition technique. J. Physics Conf. Series IOP Publ..

[23] Liu Z., Iltanen K., Chekurov N., Grigoras K., Tittonen I. (2013). Aluminum oxide mask fabrication by focused ion beam implantation combined with wet etching. Nanotechnology.

[24] Henry M.D. (2009). Alumina etch masks for fabrication of high-aspect-ratio silicon micropillars and nanopillars. Nanotechnology.

[25] Hayashi S., Yamanaka M., Nakagawa H., Kubota M., Ogura M. (1998). SiO_2_ etching using inductively coupled plasma. Electron. Comm. Jpn..

[26] Alam A.K. (2015). Etching Process Development of SiO_2_ Etching Using Inductively Coupled Plasma. Master’s Thesis.

[27] Gaboriau F., Cartry G., Peignon M.C., Cardinaud C. (2002). Selective and deep plasma etching of SiO_2_: Comparison between different fluorocarbon gases (CF_4_, C_2_F_6_, CHF_3_) mixed with CH_4_ or H_2_ and influence of the residence time. J. Vac. Sci. Technol. B Microelectron. Nanometer Struct. Process. Meas. Phenomena.

[28] Chung C.K. (2004). Geometrical pattern effect on silicon deep etching by an inductively coupled plasma system. J. Micromech. Microeng..

[29] Xu T., Tao Z., Li H., Tan X., Li H. (2017). Effects of deep reactive ion etching parameters on etching rate and surface morphology in extremely deep silicon etch process with high aspect ratio. Adv. Mech. Eng..

[30] Yeom J., Wu Y., Selby J.C., Shannon M.A. (2005). Maximum achievable aspect ratio in deep reactive ion etching of silicon due to aspect ratio dependent transport and the microloading effect. J. Vac. Sci. Technol. B Microelectron. Nanometer Struct. Process. Meas. Phenomena.

[31] Lai S., Srinivasan S., Westerman R.J., Johnson D., Nolan J.J. Notch reduction in silicon on insulator (SOI) structures using a time division multiplex etch processes. Proceedings of the MOEMS-MEMS Micro and Nanofabrication.

[32] Kim K.H., Kim S.C., Park K.Y., Yang S.S. (2011). DRIE fabrication of notch-free silicon structures using a novel silicon-on-patterned metal and glass wafer. J. Micromech. Microeng..

[33] Hong P., Guo Z., Yang Z., Yan G. A method to reduce notching effect on the anchors of a micro-gyroscope. Proceedings of the 6th IEEE International Conference on Nano/Micro Engineered and Molecular Systems.

[34] Summanwar A., Neuilly F., Bourouina T. Elimination of notching phenomenon which occurs while performing deep silicon etching and stopping on an insulating layer. Proceedings of the 2008 Ph.D. Research in Microelectronics and Electronics.

[35] Jensen S., Hansen O. (2003). Characterization of the microloading effect in deep reactive ion etching of silicon. Micromach. Microfabr. Process. Technol. IX Int. Soc. Optics Photonics.

[36] Taylor H.K., Sun H., Hill T.F., Farahanchi A., Boning D.S. (2006). Characterizing and predicting spatial nonuniformity in the deep reactive ion etching of silicon. J. Electrochem. Soc..

[37] Karttunen J., Kiihamaki J., Franssila S. (2000). Loading effects in deep silicon etching. Micromach. Microfabr. Process. Technol. VI Int. Soc. Optics Photonics.

[38] Yeom J., Wu Y., Shannon M.A. Critical aspect ratio dependence in deep reactive ion etching of silicon. Proceedings of the 12th International Conference on Solid-State Sensors, Actuators and Microsystems. Digest of Technical Papers.

[39] Blauw M.A. Deep Anisotropic Dry Etching of Silicon Microstructures by High-Density Plasmas, 2004. https://www.narcis.nl/publication/RecordID/oai:tudelft.nl:uuid:dbb050db-a834-47df-970e-208d6caf8bb3.

[40] Tang Y., Sandoughsaz A., Owen K.J., Najafi K. (2018). Ultra deep reactive ion etching of high aspect-ratio and thick silicon using a ramped-parameter process. J. Microelectromech. Syst..

[41] Farahanchi A. (2009). Characterization and Modeling of Pattern Dependencies and Time Evolution in Plasma Etching. Master’s Thesis.

[42] Slabbekoorn J., Schepers B., Gavan K.B., Sardo S., Van Huylenbroeck S., Vandeweyer T., Ranjan M. Bosch process characterization for donut TSV’s. Proceedings of the Eleventh International Wafer-Level Packaging Conference.

[43] Lai S.L., Johnson D., Westerman R. (2006). Aspect ratio dependent etching lag reduction in deep silicon etch processes. J. Vac. Sci. Technol. A Vac. Surf. Films.

[44] Abdolvand R., Ayazi F. (2008). An advanced reactive ion etching process for very high aspect-ratio sub-micron wide trenches in silicon. Sens. Actuators A Phys..

[45] Meng L., Yan J. (2015). Effect of process parameters on sidewall damage in deep silicon etch. J. Micromech. Microeng..

[46] Saman A.M., Furumoto T., Ueda T., Hosokawa A. (2015). A study on separating of a silicon wafer with moving laser beam by using thermal stress cleaving technique. J. Mater. Process. Technol..

[47] Lee J.H., Nam-Seung K., Jung-Hee L. (2011). Development of chip separation technique for InGaN-based light emitting diodes. IEEE J. Q. Electron..

[48] Haupt O., Schuetz V., Schoonderbeek A., Richter L., Kling R. (2009). High quality laser cleaving process for mono-and polycrystalline silicon. Laser Based Micro Nanopackaging Assembly III Int. Soc. Optics Photonics.

[49] Figueroa V. (2004). Designing a Mechanism to Cleave Silicon Wafers. Bachelor’s Thesis.

[50] Volosukhin V.A., Logvinov V.B., Evtushenko S.I. (1967). Strength of Materials: A Textbook.

[51] Johnson C.W., Johnson D., Martinez L., Plumhoff J. (2013). Systematic approach to time division multiplexed Si etch process development. ECS Trans..

[52] Saraf I.R., Goeckner M.J., Goodlin B.E., Kirmse K.H., Nelson C.T., Overzet L.J. (2013). Kinetics of the deposition step in time multiplexed deep silicon etches. J. Vac. Sci. Technol. B Nanotechnol. Microelectron. Mater. Process. Meas. Phenomena.

[53] Labelle C.B., Donnelly V.M., Bogart G.R., Opila R.L., Kornblit A. (2004). Investigation of fluorocarbon plasma deposition from c-C 4 F 8 for use as passivation during deep silicon etching. J. Vac. Sci. Technol. A Vac. Surf. Films.

